# Beyond the Skin: A Rare Case of Primary Malignant Melanoma of the Lung

**DOI:** 10.7759/cureus.96502

**Published:** 2025-11-10

**Authors:** Sehrish Sarwar Baloch, Vadim Zarubin, Abdul Wasio, Danial Khan Hadi, Saqib Raza Khan

**Affiliations:** 1 Department of Medical Oncology, Harold Leever Regional Cancer Centre, Waterbury, USA; 2 Department of Internal Medicine, Saint Mary's Hospital, Waterbury, USA; 3 Department of Medical Oncology, Schulich School of Medicine and Dentistry, London, CAN; 4 Department of Medical Oncology, Verspeeten Family Cancer Centre, London Health Sciences Centre, London, CAN

**Keywords:** immune checkpoint inhibitors, primary pulmonary melanoma, rare tumors, s100 protein, thoracic oncology

## Abstract

Primary malignant melanoma of the lung (PMML) is an exceedingly rare neoplasm. Due to its rarity and the high prevalence of metastatic melanoma to the lungs, PMML presents a significant diagnostic challenge and requires rigorous exclusion of extrapulmonary primary sites. We report the case of an 84-year-old lady who presented with a two-month history of fever, non-productive cough, and unintentional weight loss. Imaging revealed a solitary hypermetabolic mass in the superior segment of the left lower lobe. Histopathological examination of a transbronchial biopsy demonstrated immunohistochemical positivity for S100 and SOX10, consistent with a diagnosis of melanoma. A comprehensive dermatologic, mucosal, ophthalmologic, and systemic evaluation revealed no evidence of a primary lesion elsewhere, thereby fulfilling the criteria for PMML. Molecular testing revealed no actionable mutations (BRAF, NRAS, c-KIT), precluding targeted therapy. Given the tumour’s unresectable nature due to proximity to critical mediastinal structures and the patient’s comorbidities, she was initiated on a modified combination immune checkpoint inhibitor regimen with nivolumab and ipilimumab. PMML is a rare clinical entity with a limited number of reported cases. Diagnosis relies on histopathology, immunoprofiling, and thorough exclusion of extrapulmonary primaries. Treatment strategies are extrapolated from studies in cutaneous and unknown primary melanoma, with immune checkpoint inhibitors serving as the cornerstone of therapy in BRAF-wild-type, unresectable cases. This case highlights the importance of a meticulous diagnostic approach to solitary pulmonary lesions and underscores the evolving role of immunotherapy in managing rare malignancies such as PMML. Reporting such cases enhances the clinical understanding and guides future management of this rare disease.

## Introduction

Malignant melanoma is a neoplasm that most commonly arises from melanocytes in the skin [1]. Globally, malignant melanoma remains an important, although geographically heterogeneous, cancer burden, with an estimated 331,700 new melanoma diagnoses and 58,700 deaths globally [2]. The overall five-year relative survival for malignant melanoma, driven largely by cutaneous primaries, is approximately 62% in population-based analyses. However, in rare instances, it can originate from atypical anatomical sites such as the oral cavity, larynx, esophagus, cervix, ovaries, liver, and respiratory tract [3]. Among these, primary malignant melanoma of the lung (PMML) is sporadic, accounting for less than 0.01% of all primary pulmonary tumors, with fewer than 100 cases reported in the literature to date [4,5].

The diagnosis of PMML presents with a significant clinical challenge due to its rare nature and the necessity of ruling out more common metastatic melanoma from a cutaneous or mucosal primary. A meticulous diagnostic workup, including histopathological evaluation, immunohistochemistry, and thorough clinical and radiological assessment, is essential to exclude extracutaneous primary sites.

In this report, we present the case of an 84-year-old lady who was found to have a solitary pulmonary mass. The case highlights the diagnostic complexity of PMML and the importance of a comprehensive approach in evaluating unusual presentations of melanoma.

## Case presentation

An 84-year-old lady presented to the outpatient clinic with a two-month history of fever, persistent cough, and weight loss. The symptoms were insidious in onset and gradually worsened over time. There was no associated syncope, palpitations, orthopnea, or lower limb edema reported at the time of presentation. The patient has a significant past medical history, including coronary artery disease (CAD) with a history of coronary artery bypass grafting (CABG), hypertension, hyperlipidemia, and monoclonal gammopathy of undetermined significance. Her social history is notable for being a former smoker, having quit approximately 50 years ago. She denies any current or past alcohol use, and there is no history of vaping. There was no known family history of malignancy.

**Figure 1:**
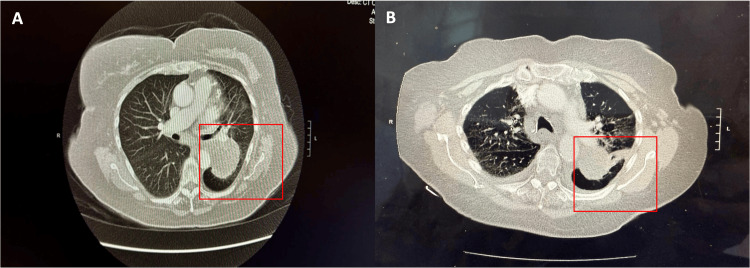
High-resolution contrast-enhanced CT scan of the chest (axial) demonstrating a large mass in the superior segment of the left lower lobe, measuring 6.0 × 5.7 × 5.8 cm (A), without evidence of additional pulmonary nodules, pleural effusion, or significant mediastinal lymphadenopathy. Repeat CT scan chest post two cycles of immunotherapy demonstrates a decrease in size of the mass in the superior segment of the left lower lobe from 5.6 cm to 4.1 cm (B) with some interstitial prominence.

On physical examination, vital signs were stable, and the overall exam was unremarkable. Initial laboratory investigations, including complete blood count, serum electrolytes, liver and renal function tests, were within normal limits. As part of the diagnostic workup, a chest X-ray was performed, which revealed a left hilar mass measuring approximately 6 cm in diameter, along with mild cardiomegaly and post-surgical changes consistent with a history of coronary artery bypass grafting. Pulmonary function tests (PFTs) were suggestive of mildly reduced diffusion capacity for carbon monoxide (DLCO). A subsequent high-resolution contrast-enhanced computed tomography (CT) scan of the chest showed a large mass located in the superior segment of the left lower lobe, measuring 6.0 × 5.7 × 5.8 cm. No additional pulmonary nodules, pleural effusion, or significant mediastinal lymphadenopathy were identified (Figure 1). A positron emission tomography (PET)-CT scan was also performed, which was suggestive of a hypermetabolic mass measuring 5.9x5.8x5.2 cm with a SUV of 21.9 in the superior segment of the left lower lobe, medially extending into the mediastinum and left hilum with significant narrowing of the left lower lobe bronchus proximally. The left descending interlobar artery was abutting the mass anteriorly, while the mass was abutting the left descending thoracic aorta. No evidence of distant metastatic disease was noted.

Bronchoscopy was performed with endobronchial ultrasound for tissue diagnosis. A transbronchial biopsy of the left lung mass was obtained, along with sampling of paratracheal and mediastinal lymph nodes. The histopathology findings of the lung lesion revealed features consistent with a high-grade malignant neoplasm. The tumor cells were predominantly spindle-shaped, with areas of tumor necrosis. Immunohistochemical staining revealed that the tumor cells were positive for S100 and SOX10, and showed focal positivity for cytokeratin AE1/AE3 and cytokeratin OSCAR. The tumor was negative for Epcam, TTF-1, p40, GATA3, CD45, calretinin, Melan-A, HMB-45, CD1a, CD163, and desmin.

**Figure 2:**
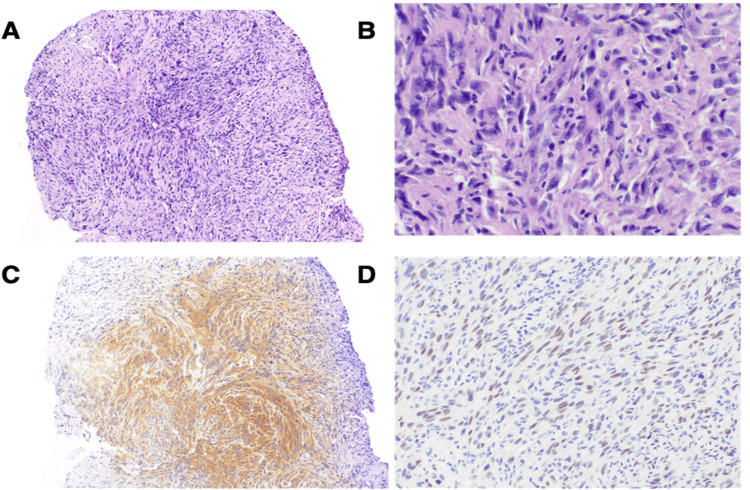
Microscopic examination of the lung tumor. A: Hematoxylin and eosin-stained section showing malignant melanoma morphology (original magnification ×10); B: Hematoxylin and eosin-stained section showing malignant melanoma morphology (original magnification ×40); C: Immunohistochemical staining demonstrating strong, diffuse positivity for S100 (original magnification ×10); D: Immunohistochemical staining showing patchy nuclear positivity for SOX10 (original magnification ×10).

The initial differential diagnosis included malignant peripheral nerve sheath tumor (MPNST), malignant melanoma, sarcoma, and carcinosarcoma. Notably, all sampled lymph nodes were negative for malignancy. Given the unusual histopathological profile suggestive of a high-grade malignant neoplasm with spindle cell morphology and immunohistochemical positivity for S100 and SOX10 the markers that are frequently expressed in neural crest-derived tumors, alongside focal cytokeratin positivity, but negative staining for a broad panel of epithelial, hematopoietic, mesothelial, and melanocytic markers, a second opinion was solicited from the pathology department at large tertiary care hospital facility, where, upon expert review and correlation with immunoprofile and morphology, the diagnosis was revised to malignant melanoma with tumor cells showing diffuse strong positivity for S100 and SOX10 (Patchy) while remainder were negative and non-contributory. Immunostains performed showed patchy weak positivity for PRAME, while BRAF V600E and NRAS Q61R were negative (Figure 2).

To further delineate whether the identified lesion represented a primary pulmonary melanoma, as opposed to a more common metastatic deposit from a cutaneous or mucosal primary, the patient was referred to dermatology for a comprehensive skin and mucosal examination, which failed to reveal any suspicious pigmented lesions, scars, or mucosal involvement suggestive of a regressed or existing primary melanoma elsewhere.

The intense FDG uptake isolated to the known left lower lobe pulmonary mass without evidence of regional or distant metastatic disease, including cutaneous, mucosal, ocular, gastrointestinal, or lymphatic sites, fulfilled the clinical and radiologic criteria required for the diagnosis of primary malignant melanoma of the lung (PMML). 

**Figure 3:**
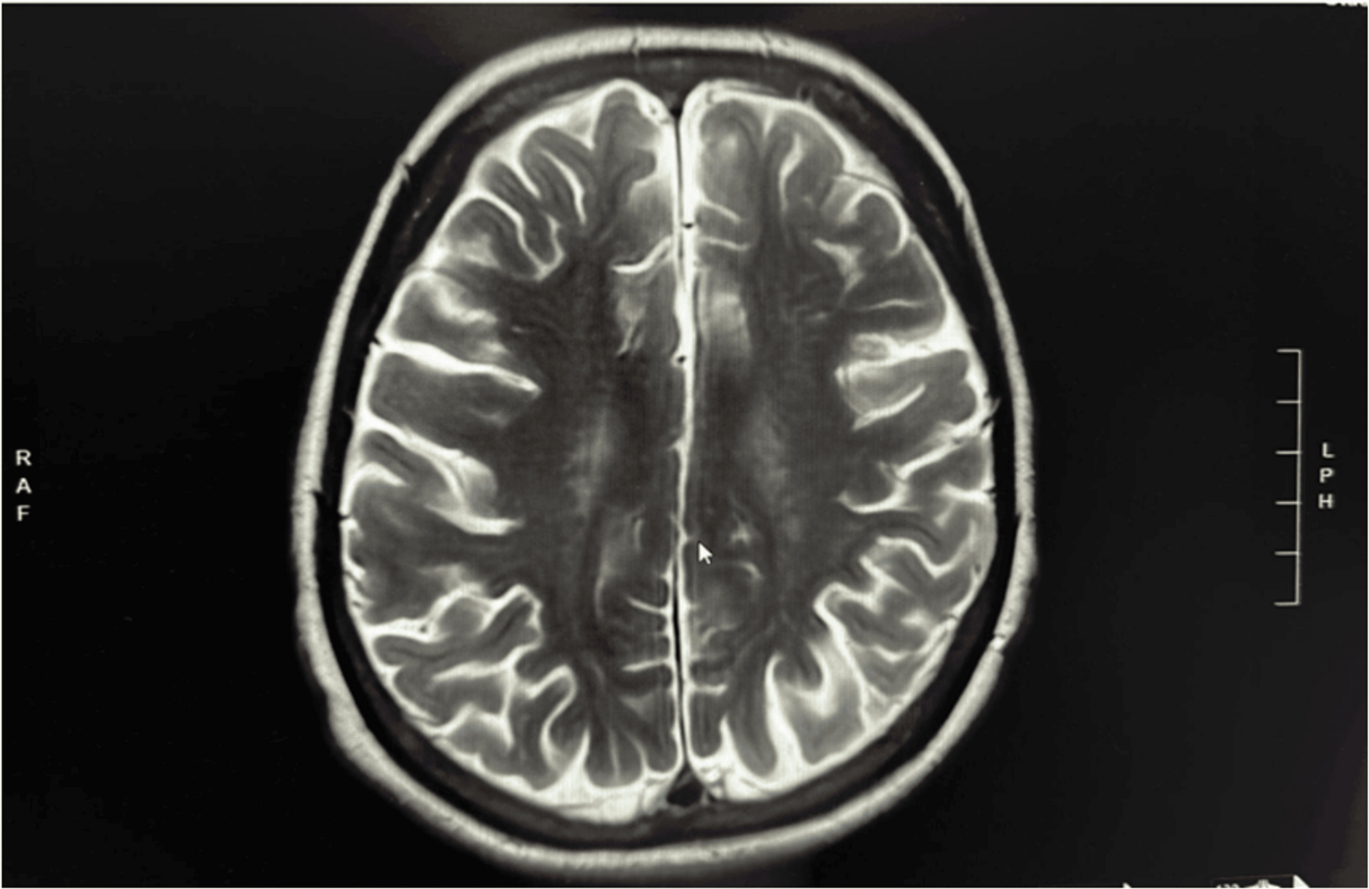
Contrast-enhanced MRI of the brain demonstrates mild chronic small vessel ischemic changes without evidence of acute infarction, parenchymal lesions, or contrast-enhancing masses, effectively ruling out intracranial metastasis.

At the time of diagnostic confirmation, the patient reported persistent constitutional symptoms, including profound fatigue, an ongoing non-productive cough, and unintentional weight loss. To evaluate for central nervous system involvement, given the high metastatic potential of melanoma, a contrast-enhanced MRI of the brain was performed. This revealed only mild chronic small vessel ischemic changes without evidence of acute infarction, parenchymal lesions, or contrast-enhancing masses, effectively ruling out intracranial metastasis (Figure 3).

In light of the lesion’s anatomical proximity to critical mediastinal structures, including direct abutment of the descending thoracic aorta and left main bronchus, as well as the patient’s significant cardiovascular comorbidities and advanced age, a multidisciplinary evaluation was undertaken, including formal consultation with the cardiothoracic surgery team. After a comprehensive assessment, the lesion was deemed unresectable due to both its locally advanced nature and high perioperative risk, precluding curative surgical intervention.

Given the patient’s progressively worsening constitutional and respiratory symptoms, including fatigue, persistent non-productive cough, weight loss, and considering her relatively preserved functional status despite advanced age, systemic therapy was initiated. The patient was commenced on a modified combination immune checkpoint inhibitor regimen, consisting of nivolumab at 3 mg/kg and ipilimumab at 1 mg/kg, administered every three weeks. Although the standard dosing of the combination therapy is nivolumab 1 mg/kg plus ipilimumab 3 mg/kg every three weeks, we recommended alternative dosing per the CheckMate 511 study, which reported reduced toxicity and hence fewer grade ≥3 adverse events compared with a standard dosing strategy. This regimen was planned for an initial two-cycle treatment followed by repeat imaging. The decision to employ a modified dosing schedule was made to balance therapeutic efficacy with tolerability in an elderly patient population; it is also supported by trial results and is in accordance with NCCN melanoma guidelines [6]. In parallel, comprehensive molecular profiling of the tumor tissue was performed to identify potentially actionable genomic alterations; however, no targetable mutations (e.g., BRAF, NRAS, c-KIT) were identified, thereby limiting the use of targeted therapies.

Following two cycles of immune checkpoint inhibitor therapy, a repeat CT scan of the chest demonstrated a reduction in the size of the left lower lobe mass from 5.6 cm to 4.1 cm, consistent with a favourable treatment response. However, new findings were noted, including ground-glass opacities, a subpleural nodular density, and a mild pleural effusion. These radiologic changes raised concern for immune-related pneumonitis versus infectious etiology (Figure 1). The patient was initiated on oral prednisone at a dose of 25 mg daily, with subsequent symptomatic improvement. A gradual steroid taper was recommended, and plans were made to proceed with the third cycle of immunotherapy.

The patient’s subsequent management strategy, including continuation of immunotherapy, consideration of local palliative measures, or supportive care, will be determined based on both the clinical response and radiologic findings following further cycles of immunotherapy.

## Discussion

Melanoma is a malignant tumor that arises from melanocytes, with more than 90% of reported cases originating in the skin [7]. Pulmonary melanomas are typically metastatic, and the occurrence of a true primary lesion in the lung is exceedingly rare [8]. Nevertheless, among extracutaneous melanomas, the lung represents the most frequent site of involvement [9]. The first cases of primary pulmonary melanoma were described by Todd in 1888, although the reports lacked sufficient documentation, leaving the true primary nature of these lesions uncertain [10].

PMML most often presents with non-specific respiratory symptoms, cough, hemoptysis, dyspnea, chest pain, or constitutional symptoms such as weight loss and fever, and radiologically as a solitary pulmonary mass or endobronchial lesion. Because such symptoms overlap with common primary lung cancers and infectious processes, PMML is frequently misdiagnosed until tissue diagnosis is obtained. Case series and reviews report that many patients present with a single dominant pulmonary lesion, and that regional or distant spread may be absent at diagnosis in a minority of cases [4]. In our patient, the presentation with months of cough, fever, weight loss, and a solitary hypermetabolic left lower-lobe mass mirrors the typical pattern reported for PMML, supporting the clinical diagnostic workup performed [7].

Furthermore, the pathogenesis of PMML remains uncertain, with prevailing hypotheses including the aberrant migration of melanocytes to the respiratory tract during embryogenesis, the malignant transformation of melanocytes within bronchial submucosal glands, or the neoplastic differentiation of pluripotent respiratory stem cells [11].

Histologically, PMML demonstrates variable morphologies, ranging from spindle to epithelioid patterns, sometimes with melanin deposition. Immunohistochemistry is indispensable, with diffuse positivity for S100 and SOX10 supporting a melanocytic lineage, while the absence of epithelial, mesothelial, and hematopoietic markers helps exclude mimics [12]. In our patient, the tumor exhibited a spindle cell morphology with necrosis, accompanied by diffuse strong S100 and patchy SOX10 expression. Staining for common epithelial, mesothelial, lymphoid, and melanocytic markers (including HMB-45 and Melan-A) was negative. Although this immunoprofile is atypical due to the absence of HMB-45 and Melan-A, the retained S100 and SOX10 expression supports melanocytic differentiation. These markers are recognized as the most sensitive indicators of melanocytic lineage, particularly in spindle cell and desmoplastic variants of melanoma, where HMB-45 and Melan-A expression may be absent [13]. The tumor was negative for BRAF and NRAS mutations, findings that guided the decision to initiate immune checkpoint blockade as first-line therapy.

The mutational profile of melanoma differs by anatomic subtype. In cutaneous melanoma, activating BRAF mutations (predominantly V600E/K) are commonly reported in approximately 40-56% of contemporary series, and NRAS mutations occur in 15- 30%. By contrast, mucosal and other non-cutaneous melanomas have lower rates of BRAF and NRAS mutations and higher frequencies of KIT and other alterations. These differences are clinically important because BRAF-V600 mutations permit effective targeted therapy, whereas many extracutaneous melanomas are BRAF-wild type and management relies primarily on immune-based strategies [14]. This is consistent with our molecular testing; no actionable BRAF/NRAS/c-KIT alterations were detected, supporting the selected immunotherapy approach.

The most widely accepted diagnostic framework for primary pulmonary melanoma with six major criteria: (1) no history of a previously removed cutaneous melanoma, (2) no history of a previously removed ocular melanoma, (3) a solitary pulmonary tumor, (4) histology compatible with a primary melanoma, (5) no evidence of melanoma in other organs, and (6) autopsy confirmation excluding a primary lesion elsewhere, particularly in the skin or eyes [15].

Our case had no history of prior cutaneous or ocular melanoma, presented with a single hypermetabolic left lower lobe mass without additional pulmonary nodules, and biopsy confirmed morphology and immunohistochemistry consistent with melanoma. Comprehensive dermatologic and mucosal evaluation, as well as PET-CT and MRI brain imaging, revealed no evidence of an alternative primary site. Although criterion six (autopsy confirmation) could not be assessed, the clinical and radiologic findings strongly support the diagnosis of PMML.

Furthermore, there are no standardized treatment guidelines for PMML. Complete surgical resection offers the best survival outcomes when feasible, but many cases are unresectable due to local invasion or comorbidities. Immune checkpoint inhibitors (ICI) targeting PD-1 and CTLA-4 have transformed the management of advanced melanoma and were chosen for this patient. Most of the available evidence for extracutaneous melanoma is extrapolated from large randomized studies conducted in cutaneous and, to a lesser extent, unknown primary melanoma. Conventional cytotoxic chemotherapy historically produced low objective response rates (10-20%), short median survival of only 6-9 months, and almost no durable complete remissions [16]. In contrast, ICIs improve objective response, prolong both progression-free survival (PFS) and overall survival (OS), and uniquely generate a subset of long-term survivors. KEYNOTE-006 demonstrated a sustained 10-year OS advantage for pembrolizumab over ipilimumab (34.0% vs 23.6%), with the majority of patients having cutaneous primaries and a minority with unknown primaries [17]. CheckMate-066 established that nivolumab was superior to dacarbazine in previously untreated, BRAF-wild-type melanoma, reporting five-year OS of 39% versus 17%, mainly in cutaneous cases [16]. Long-term data from CheckMate-067 show the greatest benefit with combined nivolumab plus ipilimumab, with a median OS of 71.9 months compared to 36.9 months for nivolumab alone and 19.9 months for ipilimumab. Most participants had cutaneous melanoma, with extracutaneous subtypes being rare. CheckMate-511 demonstrated that the “flip-dose” regimen (nivolumab 3 mg/kg + ipilimumab 1 mg/kg) achieved similar efficacy with fewer grade 3-5 toxicities compared to the standard dosing, supporting its use in frail or elderly patients, such as ours [18].

More recently, RELATIVITY-047 demonstrated that the dual LAG-3/PD-1 blockade (nivolumab + relatlimab) nearly doubled median PFS compared to nivolumab alone (10.1 vs 4.6 months), with consistent benefit in the BRAF-wild-type subgroup [19]. Collectively, these pivotal trials established ICIs as the preferred first-line approach over chemotherapy. In BRAF-wild-type diseases, such as our patient’s PMML, where targeted therapy is not applicable, they represent the most rational systemic strategy.

Finally, in the resectable setting, the Phase 3 NADINA trial demonstrated that neoadjuvant nivolumab plus ipilimumab significantly improved 12-month event-free survival to 83.7% compared to 57.2% (HR 0.32), and achieved a pathologic response in 59% of patients, establishing the benefit of early immunotherapy even in high-risk disease [20]. This case highlights both the diagnostic complexity and therapeutic challenges of PMML, reinforcing the need for multidisciplinary care and reporting of such rare cases to expand the collective understanding of this entity.

Given the rare biology and poor historical outcomes of PMML, management benefits from multidisciplinary review. For unresectable PMML that is BRAF-wild type, as in this patient, a PD-1 containing regimen is rational and supported by extrapolation from large randomized trials in cutaneous melanoma. Where feasibility exists, consideration of clinical trial enrollment is appropriate. In elderly or comorbid patients, modified dosing may help balance efficacy and tolerability; however, the risks of immune-related adverse events must be carefully weighed and discussed with the patient and their family. Palliative radiotherapy for symptom control, and consideration of limited resection or debulking when it can meaningfully improve symptoms or permit subsequent multimodality therapy, remain options in selected cases.

## Conclusions

We report an extremely rare case of primary malignant melanoma of the lung in an elderly female presenting with constitutional and respiratory symptoms. Diagnosis was established after careful exclusion of extrapulmonary primaries and confirmed by histopathology, with strong positivity for S100 and SOX10 on immunohistochemistry test. This case highlights the diagnostic challenge of PMML, coupled with the importance of comprehensive clinicopathological correlation, and adds to the limited literature on this rare entity.
